# Molecular Probing of the HPV-16 E6 Protein Alpha Helix Binding Groove with Small Molecule Inhibitors

**DOI:** 10.1371/journal.pone.0149845

**Published:** 2016-02-25

**Authors:** Anne Rietz, Dino P. Petrov, Matthew Bartolowits, Marsha DeSmet, V. Jo Davisson, Elliot J. Androphy

**Affiliations:** 1 Department of Dermatology, Indiana University School of Medicine, Indianapolis, Indiana, United States of America; 2 Department of Medicinal Chemistry and Molecular Pharmacology, College of Pharmacy, Purdue University, West Lafayette, Indiana, United States of America; International Centre for Genetic Engineering and Biotechnology, ITALY

## Abstract

The human papillomavirus (HPV) HPV E6 protein has emerged as a central oncoprotein in HPV-associated cancers in which sustained expression is required for tumor progression. A majority of the E6 protein interactions within the human proteome use an alpha-helix groove interface for binding. The UBE3A/E6AP HECT domain ubiquitin ligase binds E6 at this helix-groove interface. This enables formation of a trimeric complex with p53, resulting in destruction of this tumor suppressor. While recent x-ray crystal structures are useful, examples of small molecule probes that can modulate protein interactions at this interface are limited. To develop insights useful for potential structure-based design of ligands for HPV E6, a series of 2,6-disubstituted benzopyranones were prepared and tested as competitive antagonists of E6-E6AP helix-groove interactions. These small molecule probes were used in both binding and functional assays to evaluate recognition features of the E6 protein. Evidence for an ionic functional group interaction within the helix groove was implicated by the structure-activity among the highest affinity ligands. The molecular topographies of these protein-ligand interactions were evaluated by comparing the binding and activities of single amino acid E6 mutants with the results of molecular dynamic simulations. A group of arginine residues that form a rim-cap over the E6 helix groove offer compensatory roles in binding and recognition of the small molecule probes. The flexibility and impact on the overall helix-groove shape dictated by these residues offer new insights for structure-based targeting of HPV E6.

## Introduction

There is no effective medical therapy for women and men infected with human papillomavirus (HPV). Persistent infection with specific HPV types carries a high risk of progression from pre-malignant to invasive and metastatic cervical, anogenital and oropharyngeal cancers [1, 2]. The prototype HPV associated with “high-risk” of neoplastic transformation is HPV-16, which accounts for ~50% of all cervical cancers across the world [3, 4]. The HPV E6 protein is essential for viral replication and instrumental in bypassing host cell defenses and preventing apoptosis [5, 6]. The high risk E6 protein binds to the HECT domain ubiquitin ligase, E6AP/UBE3A and this complex is responsible for ubiquitinylation of the p53 protein, a major suppressor of tumorigenesis, resulting in its degradation by the cellular proteasome [7, 8]. This effect can be reversed in HPV-driven tumor cells by reduced expression of HPV E6, which reactivates p53 expression and leads to senescence or apoptosis [9–11]. Additional cellular factors interact with HPV E6 and may be targeted for degradation [12, 13]. These significant activities make E6 a compelling target for the treatment of HPV-associated infections.

Peptide ligands for the E6 hydrophobic groove that were derived from the alpha-helical LXXLL motif of E6AP have been characterized previously. While comparatively low in binding affinities, these peptides are able to disturb E6/E6AP interaction [14, 15]. A chimeric protein that contains the LXXLL motif and the PDZ motif displays much higher binding affinity when compared to the LXXLL motif alone [16, 17]. Interestingly, a novel small peptide unrelated to the E6AP binding motif, also inhibits E6 function by blocking the interaction of E6 with E6AP [18, 19]. Additional peptide-based protein-protein interaction inhibitors (PPI) have achieved some partial successes [20, 21].

**Fig 1:**
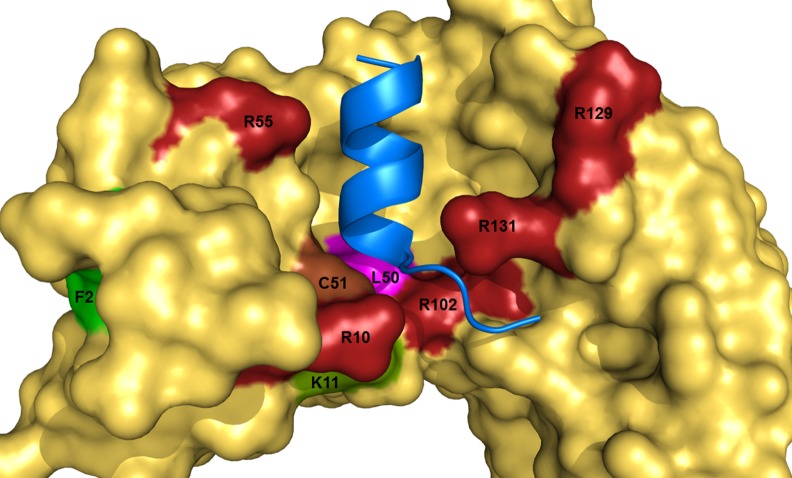
The unique topography of the α-helix binding groove of HPV-16 E6 is essential in maintaining strong polar contacts with the E6 binding motif. The rim arginines **R10, R55, R102, R129,** and **R131** form multiple hydrogen bonds with both backbone and side-chain atoms. Other key highlighted residues are **K11,** and **L50**, which are proposed to be crucial for maintaining E6 shape and activity.

The LXXLL containing alpha-helix binds in a hydrophobic pocket of E6 [22]. In this regard, the specific interface of the HPV-16 E6 protein with E6AP and its other LXXLL binding partners presents significant opportunities for targeting with small molecules (Fig 1). Despite the large overall surface area (902–1005 Å) for the groove, we and others have previously identified flavonoid derived compounds as E6 inhibitory compounds [23–27]. A limited structure-activity study identified a tetrazole-substituted benzopyranone analog that antagonized HPV-16 E6 *in vitro* and had effective IC_50_ values in the lower micromolar range [25]. Initial molecular modeling studies showed that these compounds could bind within the hydrophobic groove of the E6 protein that has been shown to contact E6AP [25]. The motivation for the current study was to understand the contribution of the 2’-6’ substitution groups on the benzopyranone scaffold and to determine what features of E6 are involved. We observed increased inhibitory activity of small molecules with charged groups at position 6 as well as a higher activity for compounds with non-polar substituents at the 2 position. Based on these results, a subset of analogs was selected to probe E6 binding interactions. Molecular dynamics simulations of the HPV-16 E6 protein implicated a high degree of flexibility of charged residues along the helix groove that could dominate small molecule interactions. A focused set of mutations at these amino acids revealed important roles in defining the molecular interactions of the E6 hydrophobic groove and offered insights for future structure-guided ligand design.

## Results and Discussion

### Characterization of MBP-E6 proteins and assay validation

**Fig 2:**
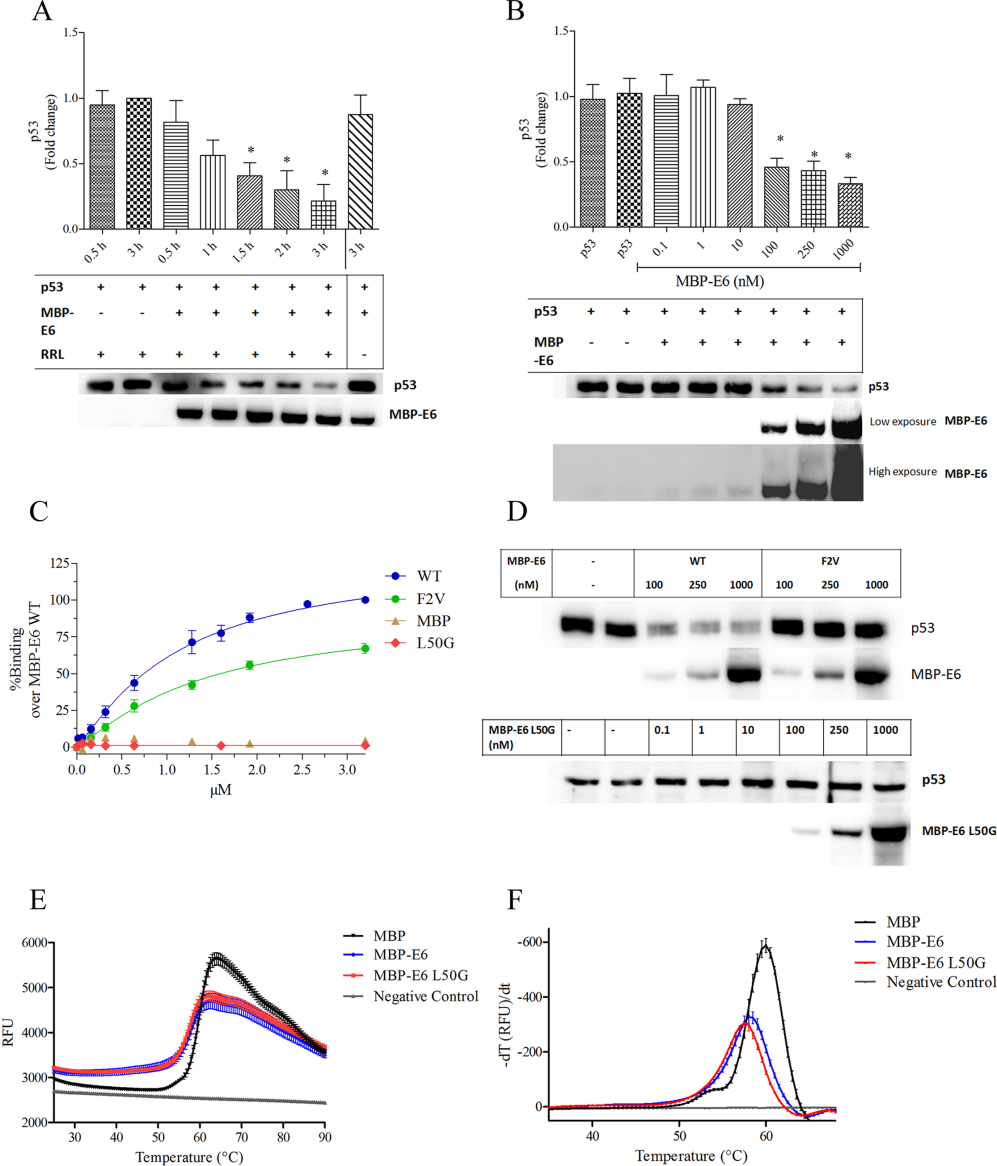
Characterization of wild-type MBP-HPV-16 E6. (A) Time course and (B) Dose response of MBP-E6 mediated p53 *in vitro* degradation activity. (C) Binding capacity of wild-type E6 and mutants F2V and L50G to E6AP in the bead-based assay. (D) p53 *in vitro* degradation with F2V and L50G. (E,F) Thermal stability profiles of MBP, MBP-E6 and MBP-E6 L50G. All data are expressed as S.E.M. *P<0.05.

To confirm the integrity of the purified MBP-HPV-16 E6 protein, we assessed its *in vitro* p53 degradation activity with respect to time and concentration (Fig 2A and 2B). The observed MBP-E6 *in vitro* p53 degradation activity was comparable to that observed for a mutant MBP-E6 in which six or four (**bold**) cysteines (C16, C51, **C80**, **C97**, **C111**, **C140**) were all replaced with serines [28, 29]. The binding capacity of MBP-E6 for the E6AP-BAP fusion protein was tested using analogous conditions previously established for GST-E6 [8, 25, 30]. For this comparison, MBP protein alone and two mutated MBP-E6 fusion proteins were included. E6 L50G, a point mutation that disables binding to E6AP and p53 [30, 31], and E6 F2V, which diminishes binding to p53 but retains partial binding to E6AP [30, 32], served as controls (Fig 2C). MBP-E6 F2V retained ~70% of the wild-type binding capacity, while MBP and MBP-E6 L50G showed no significant binding to E6AP. Neither MBP-E6 L50G, nor F2V induced degradation of p53 at the concentrations studied (Fig 2D).

A second binding assay was employed based upon MBP-E6 unfolding in the Sypro Orange- based thermal stability assay (TSA) [25, 33]. Purified MBP protein denaturation showed a Tm of 59.7 ± 0.1°C, which is comparable to wild-type MBP protein (62.3°C; pH 8.2 [34]). The slight difference in our assay (pH 6.8) is due to the reported pH sensitivity of MBP [34]. MBP-E6 fusion proteins displayed increased basal fluorescence compared to MBP alone, implying that MBP does not limit accessibility of the E6 protein to Sypro Orange (Fig 2E). Analysis of thermal stability of fusion-proteins could result in two melt peaks from unfolding of the MBP and E6 domains. Only a single peak was observed with MBP-E6 that is consistent with a predicted Tm between 55–65°C of HPV-16 E6 (Fig 2F; MBP-E6 Tm: 58.2 ± 0.1°C). The melting temperature of purified wild-type E6 alone is difficult to assess due to its aggregation-prone nature [35, 36]. However, an HPV-16 E6 mutant protein, in which all nine cysteines were converted to residues found in HPV-1A, had a Tm of ~60°C [27].

### Inhibitory compounds bind to MBP-HPV-16 E6 protein

**Fig 3:**
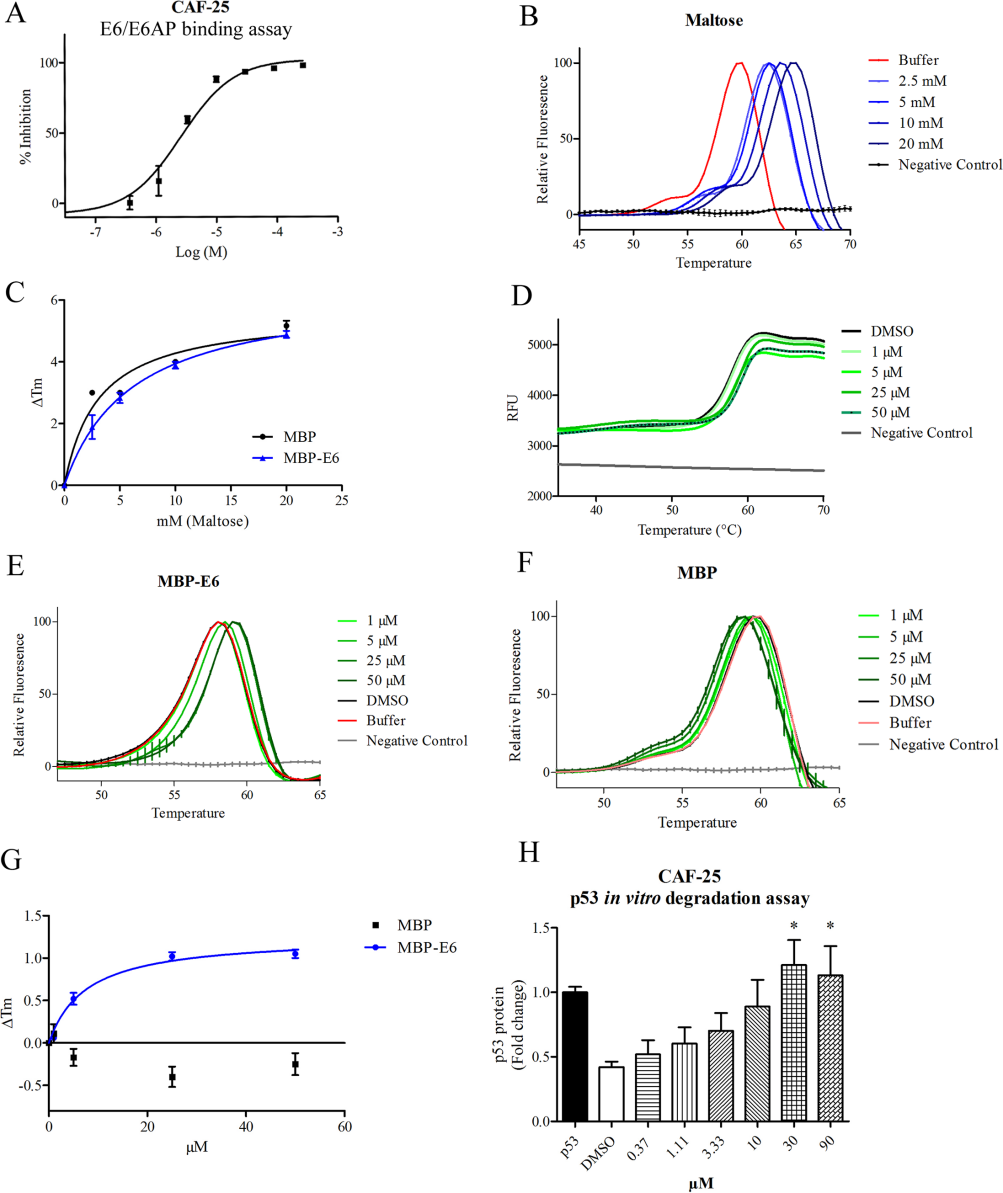
CAF-25 binding to MBP-HPV-16 E6. (A) Dose response effect of CAF-25 on MBP-E6/E6AP binding in the bead-based assay. (B) Effect of maltose on MBP melt curve in the TSA. (C) Dose dependent effect of maltose on the melting temperature changes (ΔTM) on MBP and MBP-E6. (D) Raw fluorescence plot of MBP-E6 melt curves in response to increasing CAF-25. Relative fluorescence plots of MBP-E6 (E) and MBP (F) melt curves in response to CAF-25. Negative control is composed of Sypro Orange in buffer. (G) Summary of the ΔTM changes of MBP and MBP-E6 by CAF-25. (H) Inhibition of MBP-E6 mediated p53 degradation by CAF-25. All data are expressed as S.E.M. *P<0.05.

**Fig 4:**
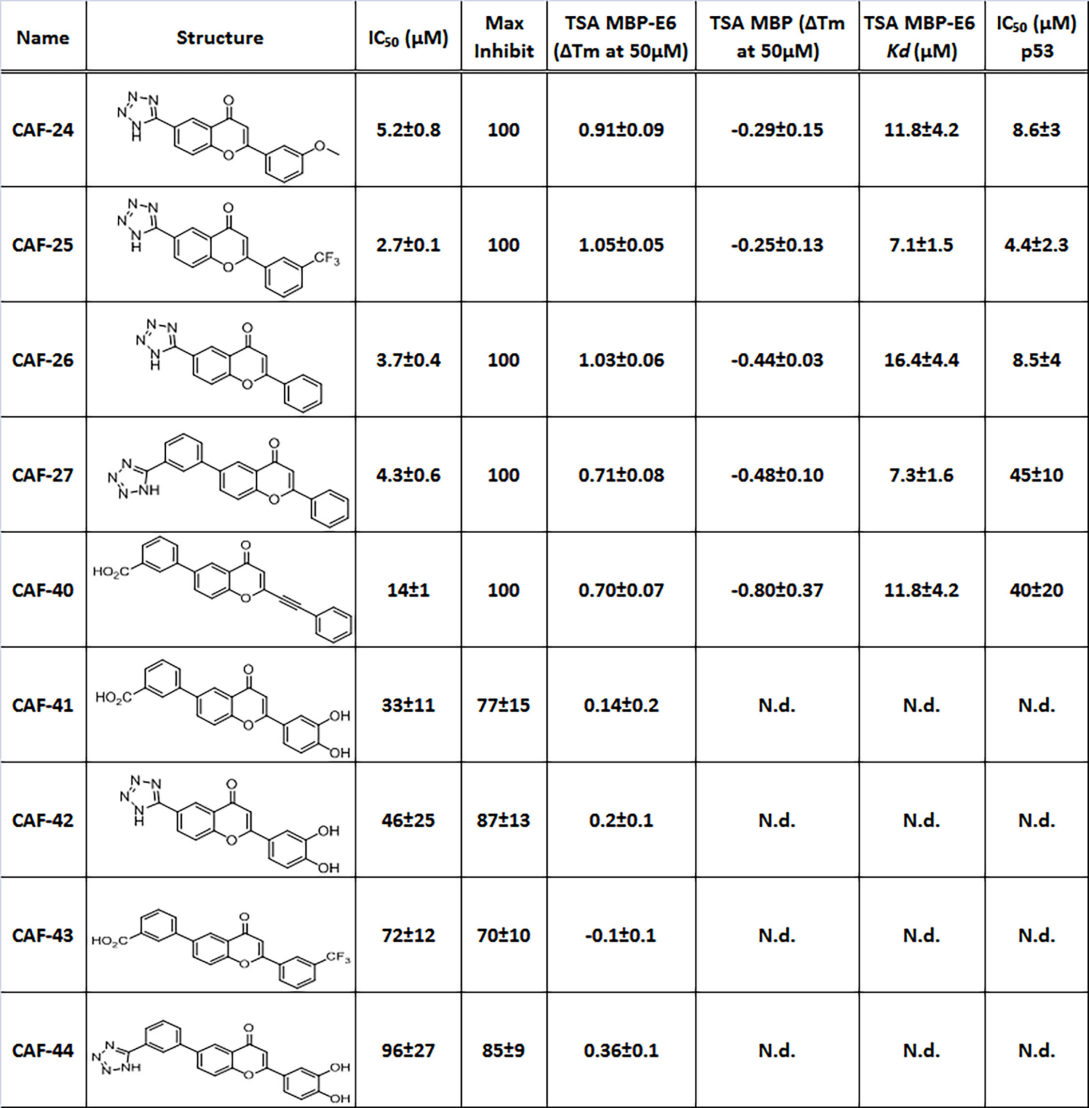
Interactions of 2 and 6 substituted benzopyranone analogs with MBP-HPV-16 E6. IC50 and maximal inhibition determined in the MBP-HPV-16 E6/E6AP binding assay; ΔTM change of MBP-E6 and MBP at 50 μM compound over DMSO control in the TSA; TSA MBP-E6—apparent *K*_*d*_ of compound interaction; IC50 in the p53 *in vitro* degradation assay with MBP-E6. N.d. not determined.

Previously, a series of carboxylate- and tetrazole-substituted benzopyranones were reported to inhibit the E6-E6AP peptide interactions [25]. The roles and contributions of the 2–6 functional groups on the benzopyranone core were investigated to gain insight regarding the potential sites of interactions with HPV E6. Compounds CAF-24, CAF-25 and CAF-26 displayed similar apparent binding with the E6AP displacement assay using MBP-E6 fusion proteins (Fig 3A, Fig 4) [25]. To further establish the TSA as a means to measure direct compound binding to HPV-16 E6, we first determined the MBP and MBP-16 E6 melt curves in response to increasing doses of maltose (Fig 3B). We observed similar ΔTm changes in response to maltose with both proteins, indicating comparable accessibility of the MBP tag (Fig 3C). The E6 inhibitor CAF-25 stabilized purified MBP-E6 in a saturation-dependent manner (Fig 3D–3G and Fig 4). MBP itself was either not affected or destabilized with these compounds, providing evidence for selective binding to the E6 protein. The destabilizing effect on MBP protein may contribute to the smaller global ΔTm changes detected with these compounds and MBP-E6 protein. In addition, the relatively large MBP-tag could mask true ΔTm changes upon compound binding to the relatively small E6 protein. We observed that at 150 μM, some of the E6 inhibitors quenched the melt curve, hence we chose to compare the ΔTm changes at 50 μM (Fig 4). Compounds that performed consistently in the binding and TSA assays were investigated using the p53 *in vitro* degradation assay (Fig 3H; S1A Fig; and Fig 4). Compounds CAF-24, -25, -26 and CAF-40 showed similar efficiencies in the binding and functional E6 mediated p53 degradation assays, while CAF-27 exhibited lower than expected efficiency. This may be due to lower binding affinity as indicated by the smaller ΔTm changes. Compounds with a tetrazole group in the 6' position of the benzopyranone core showed enhanced selectivity for the E6 protein when compared with the benzoate functional group at the same position. There also appears to be preference for non-polar substituent in positions 3 and 4 of 2-aryl group. While unsaturated ketones are prone to be pan assay interference (PAINS) compounds [37, 38], we believe that the observed interaction of tetrazole-based flavone compounds with E6 shows specificity based on the following evidence. Probes did not 1) inhibit the binding of FLAG beads to FLAG-tagged E6AP-BAP peptide, indicating no interference with BAP activity itself or interference of antibody recognition of the fusion protein, 2) inhibit interaction of hDLG with E6AP-BAP peptide [25], or 3) stabilize MBP protein in the TSA. In addition, the binding assay contains 0.1% milk to reduce non-specific interactions. While other off-target effects cannot be excluded, these results motivated the question of what key recognition features of the E6 protein are being exploited by these substitutions. Molecular docking of the 2–6 substituted benzopyranone indicated a minimal set of poses and implicate direct hydrophobic groove binding on E6. However, molecular docking analysis using Glide Induced-Fit scores did not correlate with experimental results (data not shown).

### Molecular Dynamics simulations

**Fig 5:**
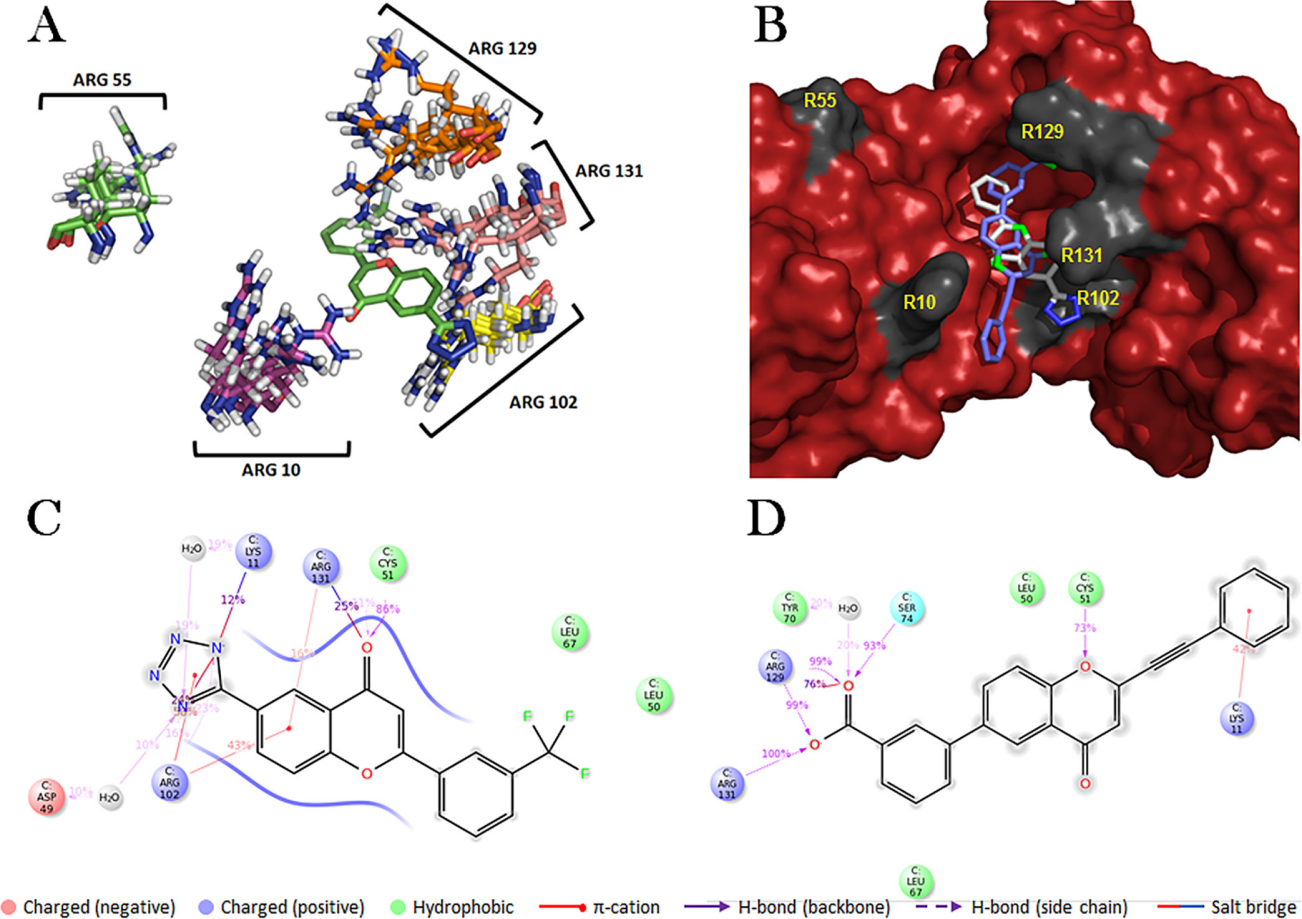
Molecular dynamics (MD) simulations of CAF-25 and CAF-40 with HPV-16 E6 (PDB ID: 4GIZ). The five arginine residues surrounding the binding groove are highly flexible (A) and both CAF-25 and CAF-40 assume similar docking orientations, maximizing their interactions with R102, R129, and R131 (B). Panels C and D show a detailed interaction map with the binding groove of CAF-25 and CAF-40, respectively. In both cases, H-bonding and π-cation contacts with R102, R129, R131, K11, C51, and S74 form the primary basis for interaction with E6.

The observed differences between experimental data and *in silico* docking results motivated an alternative approach to assess compound binding to HPV-16 E6 using molecular dynamics simulations. CAF-25 and CAF-40 were chosen for computational modeling as representative of the tetrazole and non-tetrazole classes, respectively. Each have the lowest IC_50_ values in the respective classes, and CAF-40 features an alkyne moiety with additional carbons at the position 2' of the benzopyranone core, allowing it to explore a potentially different and larger area of the binding pocket. Over the course of each simulation, it became evident that arginine residues along the periphery of the E6AP binding groove play a substantial role in the non-bonding interactions between the ligands and the protein. We observed a previously unseen flexibility of specific arginine residues that reside on the edges of the hydrophobic pocket (Fig 5A, 5B and S1C, S1D Fig) and partially define the shape of the groove. The identified interactions of CAF-25 and CAF-40 with E6 protein (PDB ID: 4GIZ) using MD simulations are summarized in Fig 5C, 5D and S1C, S1D Fig. Arginine 102 maintained contact with the CAF-25 ligand throughout the majority of the simulation time frame (Fig 5C), while cysteine C51 was found to play a major role by forming a backbone hydrogen bond with the benzopyranone ketone oxygen of CAF-25. The C51 residue also maintained contact with CAF-40; however, in this case the ring oxygen of the benzopyranone was involved (Fig 5D and S1D Fig).

### Characterization of E6 mutants

**Fig 6:**
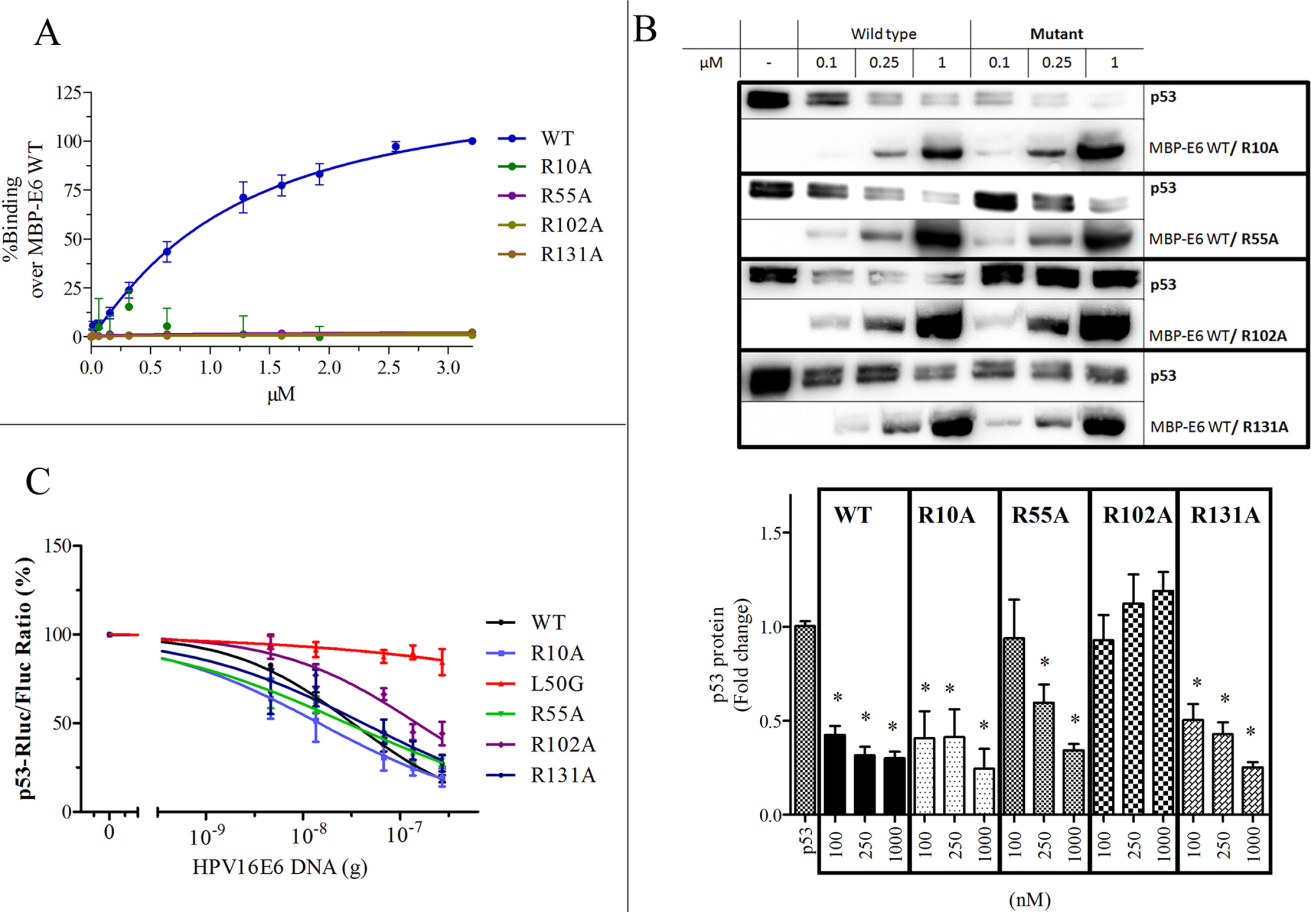
Characterization of HPV-16 E6 arginine mutants. (A) Binding of MBP-E6 wild-type and mutants R10A, R55A, R102A and R131A to E6AP in the bead-based assay. (B) p53 *in vitro* degradation by wild-type and MBP-E6 mutants. Top panel: representative western blots blotted with anti-p53 and anti-E6. Bottom panel: Densitometric analysis of p53 expressed as fold change over p53 control. (C) C33a cells transiently transfected with p53-luc, firefly luciferase and increasing DNA concentrations of 16E6 and mutants analyzed for luciferase activities at 24 hrs. All data are expressed as S.E.M. *P<0.05.

To test the implications from the MD models of compound binding, a series of point mutations of four arginines to alanine (R10A, R55A, R102A, R131A) were prepared. These E6 mutant proteins did not bind to E6AP-BAP in the bead based assay at the concentrations studied (Fig 6A). Analysis of melting curves indicated that none of the R to A mutant E6 proteins appeared to be destabilized relative to MBP-E6 wild-type (S1E Fig). In contrast, these E6 mutants demonstrated significant activity in the p53 degradation assay, with R10A and R131A maintaining activities comparable to wild-type MBP-E6 (Fig 6B). However, R55A was 50% as active and R102A was defective at the concentrations studied (Fig 6B). These *in vitro* p53 degradation activities are in agreement with previously published results for the HPV-16 E6 protein containing four cysteine to serine mutations 4C4S [39]. To clarify whether purified MBP-E6 R102A may exert p53 degradation activity if allowed to react longer, reactions were incubated for 3h at which R102A decreased p53 by 50% at 1 μM but not at 0.25 μM (S1F Fig). Further evaluation of these E6 mutants was also investigated using a cell-based assay with a p53-luciferase (p53-luc) reporter construct [40]. HPV-16 E6 wild-type decreased levels of the p53-luc protein, while the HPV-16 E6 L50G mutant did not in the range of concentrations studied. Similar to *in vitro* degradation assays, E6 mutants R10A and R131A showed activity equivalent to wild-type, while E6 R55A gained activity in this assay relative to wild-type. HPV-16 E6 R102A was partially defective in the *in vitro* assay and also appears partially defective in reducing levels of p53-luc (EC50 R102A vs. wild-type: 172±43 vs. 32±2 ng; Fig 6E). In summary, these results indicate a greater role of R102 in the interaction with E6AP than the other arginine residues studied. Notably, R102 and R55 are conserved in high risk HPV-18 E6, which also binds E6AP and induces degradation of p53. The inability to detect E6 mutant binding to E6AP-BAP *in vitro*, while these retained E6AP-dependent p53 degradation *in vitro* and in cells, may reflect the different experimental conditions or a stabilizing contribution of p53 in the trimeric complex (E6/E6AP/p53).

### Characterization of compound binding to E6 mutants

**Fig 7:**
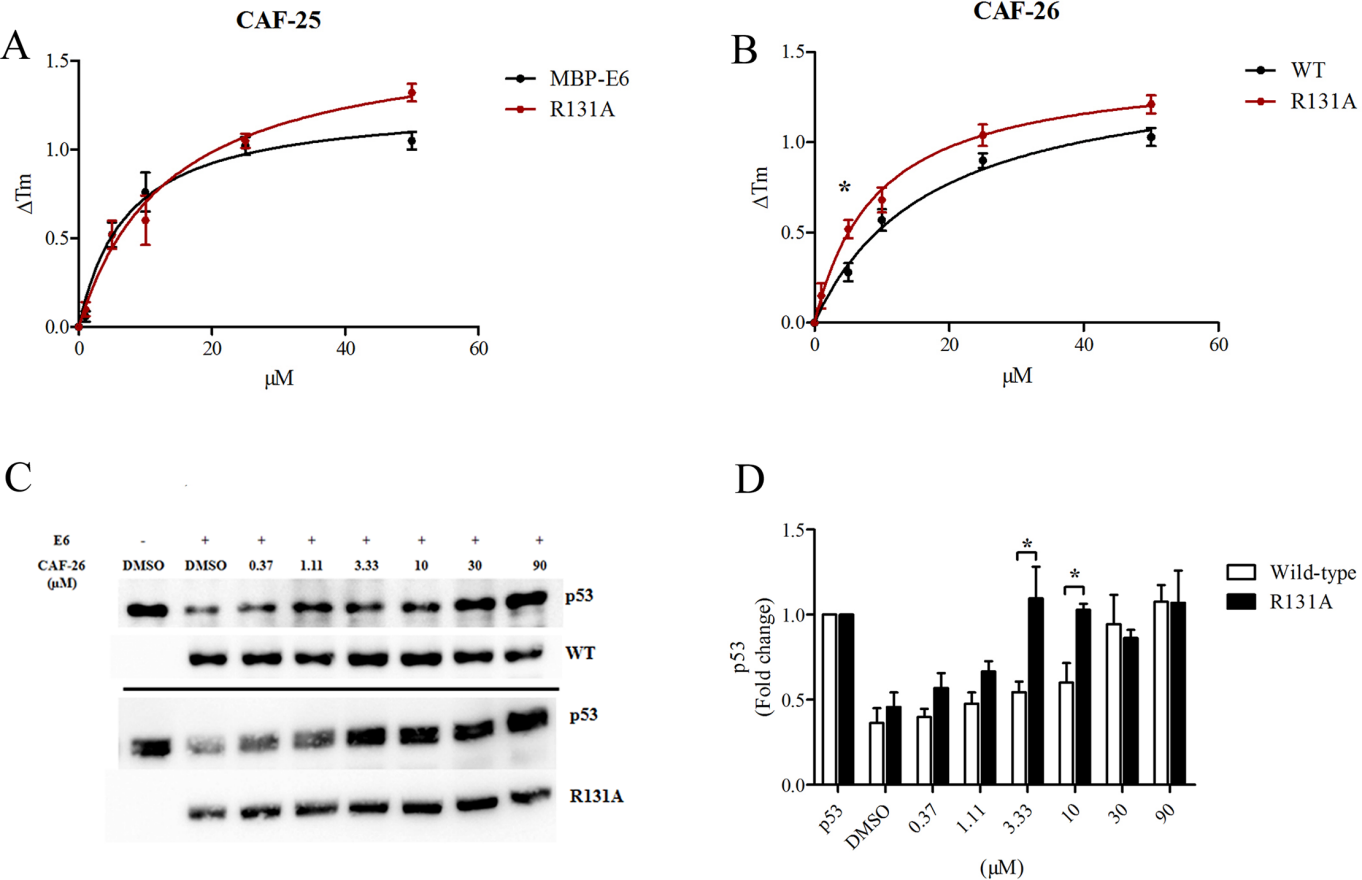
Interaction of CAF-25 and CAF-26 with MBP-E6 R131A protein. ΔTM profile of MBP-E6 and R131A in response to increasing concentrations of (A) CAF-25 and (B) CAF-26. (C) Western blot of the p53 *in vitro* degradation activity of MBP-E6 wild-type (top) and R131A mutant (bottom) in response to CAF-26. p53 protein and MBP-E6 proteins were blotted using p53 (pAB1801) and anti-E6 antibody (AVR 813). (D) Densitometric analysis of p53 protein expressed as fold change over p53 control for wild-type E6 and R131A in response to increasing CAF-26. All data are expressed as S.E.M. * P<0.05.

The contribution of the HPV-16 E6 arginine mutations to compound binding was tested in the TSA (Fig 7A and 7B, S2–S5 Figs and S2 Table). Compounds CAF-25, CAF-26, CAF-27 and CAF-40 were chosen as test molecules. The CAF-25 and CAF-26 compounds have close structural similarities except for the CF_3_ group in position 3' of the 2-aryl group. We included CAF-27 and CAF-40 as these represent elongated structures, which would occupy a larger volume of the hydrophobic-groove but also engage some ionic features with the tetrazole groups. MD simulations also indicated that the interaction pattern of CAF-25 and CAF-40 with E6 could be sufficiently different to be distinguished in the TSA.

Equilibration of R55A and R102A proteins with CAF-25 or CAF-26 resulted in a ΔTm saturation curve similar to wild-type E6 (S2 and S3 Figs). Mutant proteins R10A and L50G displayed slightly higher ΔTm shifts with CAF-25 compared to wild-type, but lacked statistical significance (S2 Fig). CAF-26 led to a significant increase in the ΔTm of R10A protein at 50 μM compared to wild-type, while L50G exhibited a saturation curve similar to wild-type (S3 Fig). CAF-25 shifted the binding curve of MBP-E6 R131A to a higher apparent *K*_*d*_ (R131A vs. wild-type: 13.5 ± 2.7 vs. 7.3 ± 2.7 μM), a result of a higher ΔTm shift at 50 μM (Fig 7A), while CAF-26 led to higher ΔTm changes of R131A even at low concentrations (Fig 7B).

Unexpectedly, the thermal denaturation profiles of the E6 mutants with CAF-27 resembled wild-type (S4 Fig). R55A showed a decreased ΔTm shift at the highest concentration of 50 μM at which saturation seemed to occur. It is possible that the elongated structure of CAF-27 is flexible and adjusts to the changes in the mutant E6 protein structure. However, due to the absence of effects across all E6 mutant proteins, it seems more likely that this compound has a lower binding dependence for the arginine residues targeted in this study.

The unanticipated flexibility of the HPV-16 E6 helix-groove interaction sites could account for the minimal impact on thermal stabilities of the arginine point mutants in combination with ligands. The observed increased thermal stabilization of R131A with CAF-25 and CAF-26 suggests that mutation to alanine provides improved steric access to the pocket, leading to increased interactions with R10 and opposed R129 in addition to stabilization by K11 as seen in wild-type. Precedence for an apparent increased binding has been reported for the E6 R129A mutation using a luciferase-based interaction assay with a novel peptide, while R129E reduced the effect [18]. To further substantiate the finding with the R131A protein, the p53 *in vitro* degradation assay was conducted in the presence of increasing concentrations of CAF-26. As shown in Fig 7C and 7D, R131A induced p53 reduction was inhibited by **~**5 times the IC_50_ of CAF-26 relative to wild-type, consistent with the TSA binding data.

The benzoic acid-based flavonoid analog CAF-40 stabilized wild-type E6 as well as the R10A, L50G and R131A mutant proteins (S5A, S5B and S5E Fig), whereas binding to R102A showed a higher calculated *K*_*d*_ (S5D Fig and S2 Table). A similar effect was observed with the R55A mutant, but the data were not statistically significant (S5C Fig and S2 Table). Since the R102A mutant has very low p53 *in vitro* degradation activity, inhibitory effects of CAF-40 were not evaluated.

These experimental results were evaluated in the context of MD simulations to provide an additional basis for understanding the relevant residues involved in the HPV-16 E6 protein-ligand interactions. As before, the simulations were executed with the CAF-25 and CAF-40 E6 antagonists. For E6, R10A, R55A and R131A proteins, R102 maintained contact with the CAF-25 ligand throughout the majority of the simulation time frame (S6 Fig). In the case where R102 was mutated to alanine, the amount of contact between R131 and the ligand increased. Furthermore, the apparent interaction of CAF-25 with C51 in E6 was found to play a major role in nearly every simulation frame of R10A, R55A and R131A, but was limited in mutants L50G and R102A. In regards to L50G, other hydrophobic residues in the E6 pocket appear to resume this interaction, and the ligand is stabilized by R10 instead of adjunct K11, and R131 instead of its opposing residue R102 on the outer surface of the pocket. The interaction of R102A with CAF-25 may induce an alternative ligand position that is stabilized by residues further outside of the pocket, in particular by interactions with I128, R129, G130 and substantially by R131 (S6D Fig).

When the experimental results were compared with the MD simulation data for CAF-40 with mutant E6 proteins (S7 Fig), it became evident that interaction between the carboxylate group and R10 was minimal, which is consistent with the TSA results (S7A Fig). Further, R55 was not observed to interact with CAF-40 in E6 MD simulations, whereas its importance increased significantly in the R10A, L50G and R131A mutant proteins (S7B and S7C Fig). E6 MD simulations also predict a negligible direct interaction between CAF-40 and R102, whereas binding data suggests R102 is important to CAF-40 binding (R102A mutant, S2 Table). This discrepancy can be explained by thorough analysis of the R102A MD results–the R102A mutation does not directly impact CAF-40 binding (S7D Fig). However, it leads to a shift in the position of R131 such that it is no longer able to participate in direct H-bonding with the carboxylate moiety, but does so through a water molecule (S7E Fig). The entire ligand shifts down, picking up a new π-cation interaction with R10, but this is insufficient to make up for the loss of direct R131 H-bonding (S7F Fig). This analysis is also supported by the distances between the R102 side chain and the carboxylate group in CAF-40 (3.4Å and 4.5Å, E6 MD), which are outside of the typical H-bond length (≈3 Å). In the case of the R131A mutant, the loss of H-bonding and π-cation contacts is so significant that CAF-40 rotates 180° to facilitate a highly-efficient interaction with R102.

Interestingly, in the MD simulations with HPV-16 E6 protein, there was a CAF-40 interaction with L50 during the entire simulation frame. However, in the L50G protein, CAF-40 was increasingly stabilized by adjacent hydrophobic residues in the pocket, as well as by R55, R102, R129, R131 on the outer surface. These compensatory features may account for the observed similar thermal profiles of L50G and wild-type. The stabilization of L50G by these E6 inhibitors is an observation as L50G fails to bind E6AP and is unable to initiate p53 degradation. Nonetheless, previous research showed that HPV-16 E6 L50G binds p300 and inhibits transcriptional down regulation of p53 [31], binds to the hTERT promoter [30], interacts with ING4 and inhibits p53 acetylation similar to wild-type E6 protein [41, 42]. Together these data indicate that the tested tetrazole-based compounds have greater flexibility than CAF-40 in binding to the E6 protein.

**Fig 8:**
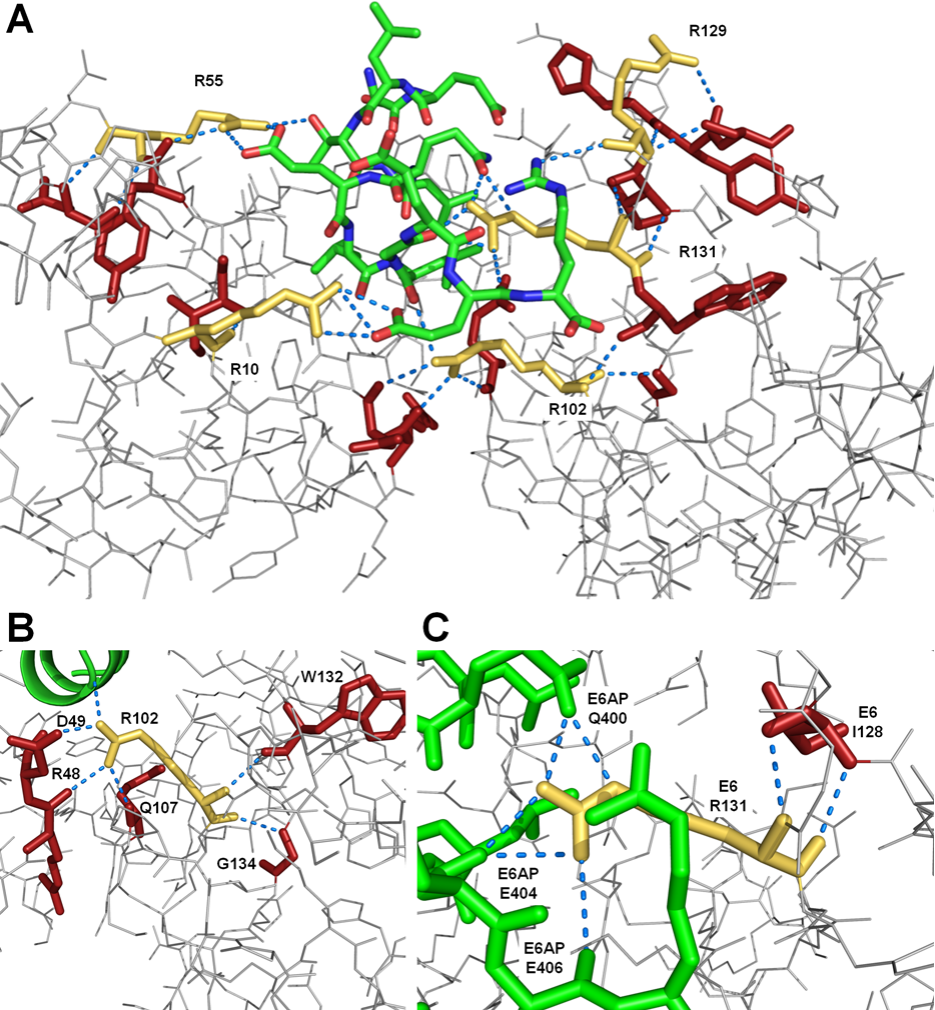
The rim arginines R10, R55, R102, R129, and R131 play a major role in the shape of the E6 helical binding groove, as well as in molecular recognition of the binding partners. (A) The arginine residues (yellow-gold color) form multiple polar contacts (blue dotted lines) with E6AP (green backbone), which are proposed to contribute to its binding affinity and alpha-helical shape (B,C). Concurrently, the rim arginines also form important intra-molecular polar contacts (blue doted lines; all HPV-16 E6 residues involved in polar contacts with the rim arginines are colored red), which are believed to shape the floor and sides of the groove. R102 is important for groove floor formation (B), while R131 plays a role in forming the right side of the binding pocket (C).

Structure-guided approaches to design small molecule antagonists for helix-groove protein-protein interactions have been successfully deployed in a few cases such as MDM2-p53 [43]. A structure-based design for discovery of HPV-16 E6 antagonists will require consideration of the unique features of the hydrophobic groove. Since the HPV-16 E6 target specificity allows degeneracy, the challenge of understanding what mediates the primary protein-protein interactions could partially be explained by the attributes of compensatory roles of charged side chain residues revealed in these studies. More importantly, the role that these residues play in forming the shape of the helix groove is now observed to be more critical. In Fig 8A are highlighted examples of how the rim arginines relate to the non-hydrophobic volume that defines the E6 protein, when occupied with an alpha helical binding partner. Fig 8B and 8C shows the extensive network of charged and hydrogen binding interactions that relate to trans-groove interactions and maintain helical partner protein contacts. The two examples of R131 and R102 are particularly noteworthy, given how the non-bonding interactions form the bottom of the groove and likely stabilize the alpha helical features of the peptide.

These data highlight the benefit of combined experimental and molecular dynamics modeling approaches to gain perspective into the types of molecular interactions that contribute to binding in the hydrophobic groove of HPV-16 E6. While the original concept of developing a pharmacophore based upon a hydrophobic patch in the E6 helix-binding groove provided the first class of inhibitors, the nature of the ligand interactions are different than expected [25, 44]. From exploration of the benzopyranone scaffold, there are important features of E6 that have been revealed, which can impact future design strategies for E6 inhibitory compounds. The results here indicate that considerations of both the flexibility as well as the electrostatic features of the helix binding groove will be useful. These observations are interesting in light of recent roles of electrostatic interactions in the rim of the MDM2 alpha-helix binding groove used to design more potent and selective inhibitors [45]. Unlike MDM2, a hotspot in the hydrophobic floor of the E6 groove has not been easily identifiable for specific inhibitor design [46]. More likely, the hydrophobic groove contacts accommodate more degeneracy in molecular recognition of target proteins given the multiple binding partners and diverse biological activities of E6.

## Methods

### Cloning and site-directed mutagenesis

MBP-HPV-16 E6 cloned in pETM-41 was a kind gift of G. Travé. Mutations of MBP-HPV-16 E6 as well as pLXSN HPV-16 E6 were performed using Finnzymes' Phire® Hot Start DNA Polymerase (Thermo Scientific). The MBP control vector was generated by inverse PCR using 5'-end phosphorylated Primers and Phire Hot Start polymerase (S1 Table). Primers used for the site-directed mutagenesis are summarized in S1 Table.

### Protein expression and purification

Protein purification of MBP, MBP-HPV16 E6 and its mutants was performed as described [25] with the following variations. In brief, plasmids were transformed in BL21 (DE3) *E*. *coli* and clones were grown in 10 ml of TurboBroth^TM^ (AthenaES) supplemented with 0.04% (w/v) glucose. Protein stocks were prepared as 2 ml frozen *E*. *coli* aliquots (20% (v/v) glycerol). For protein production 2 ml of the bacterial stock was inoculated with 200 ml Turbo-broth supplemented with 0.05% (w/v) glucose, 0.2% (w/v) lactose, 0.6% (v/v) glycerol and incubated at 20°C, 200 rpm for 30 h. Cells were collected at 6000xg, 15 min, 4°C. The pellet was resuspended in 400 mM NaCl, 50 mM Tris-HCl pH 6.8, 0.1% (v/v) NP-40, 2.5 mM DTT and 1x protease inhibitor cocktail (EDTA-free; Sigma-Aldrich). The cells were sonicated and suspension was cleared at 15,000x g, 4°C for 30 min. The supernatant was incubated with amylose beads (NE Biolabs) rotating at 4°C for 2h. The beads were washed once with lysis buffer in 10 times the bed volume. Subsequent washes were performed with lysis buffer without NP-40. Proteins on beads were collected in storage buffer (400 mM NaCl, 50 mM Tris-HCl pH 6.8, 2 mM DTT, 1x protease inhibitor) or eluted with Maltose at a final concentration of 10 mM for 1h rotating at 4°C. Eluted proteins were cleared by centrifugation at 3000xg, 4°C, 5 min. Protein concentration was determined by Bradford assay and were diluted to 1 μg/μl with storage buffer containing 10 mM maltose.

E6AP-BAP (pET-24a) was transformed into BL21(DE3) *E*. *coli*. Clones were picked and inoculated in 10 ml of TurboBroth supplemented with 0.04% (w/v) glucose. Protein stocks were prepared as 1 ml frozen *E*. *coli* aliquots (20%(v/v) glycerol). For protein production, 2ml of the bacterial stock was inoculated with 300 ml Turbo-broth supplemented with 0.05% (w/v) glucose, 0.2% (w/v) lactose, 0.6% (v/v) glycerol and incubated at 37°C, 200 rpm for 4h followed by 24h at 20°C, 200 rpm. Cells were collected as described earlier and resuspended in 100 mM NaCl, 100 mM Tris-HCl pH 8.0, 0.1% (v/v) NP-40, 0.5 mM PMSF and 1x protease inhibitor followed by sonication as described above. Supernatant was incubated with 200 μl anti-flag resin (50% slurry, Sigma-Aldrich) for 4h, rotating, 4°C. This mixture was transferred to an EconoColumn and washed with 10 bed volumes of 100 mM NaCl, 100 mM Tris-HCl, 0.1% (v/v) NP-40 and 1x protease inhibitor. E6AP-BAP was eluted with 5 successive elutions of ice-cold 0.1 M glycine pH 2.0 into 250 μl of Neutralization buffer (50% (v/v) glycerol, 200 NaCl, 200 mM Tris-HCl pH 8.0, 2x protease inhibitor cocktail). All bacterial expressed proteins were stored at 4°C and used within 5 days after purification. Human p53 proteins were expressed in High 5 cells using human p53 baculovirus that was a kind gift from Carol Prives. Cells were rinsed with PBS and incubated with lysis buffer (50 mM Tris-HCl pH 6.8, 400 mM NaCl, 0.4% (v/v) NP-40, 2 mM DTT) and 1x protease inhibitor cocktail (EDTA-free). Lysate was cleared by centrifugation at 20,000xg and diluted in lysis buffer 1:200. P53 protein levels were evaluated by immunoblotting using D0-1 and PAB1801 antibody.

### p53 degradation assays

*In vitro* p53 degradation assays were performed in a 30 μL reaction in assay buffer (25 mM Tris HCl pH 7.5, 100 mM NaCl, 2 mM DTT, 2.5 mM ATP) containing 1 μl rabbit reticulocyte lysate (RRL), p53, E6 proteins or E6 buffer. Reactions were incubated for 2h at 25°C unless otherwise stated and were stopped by incubation at 75°C. For inhibition studies, E6 proteins were diluted to 0.1μM and equilibrated with assay buffer containing 0.35% (v/v) DMSO, or compounds at different concentrations for 1h, followed by the addition of p53 and RRL and incubation at 25°C for 2h. Proteins were separated on 10% SDS page, transferred onto PVDF membrane and blotted for p53 using Pab1801 antibody. Blots were stripped and re-probed with anti-HPV-16 E6 antibody (ArborVita Inc.). All p53 degradation experiments were repeated at least three independent times.

The p53 *in vivo* degradation assay was performed as described with the following modifications [40]. C33A cells were transfected with pRluc-p53 (215 ng), pCI-Fluc (135 ng), HPV-16 E6 expression vectors (0, 18.5, 54, 270, 540, 1080 ng) and carrier DNA (up to 1080 ng, pBabePuro) using Lipofectamine 2000 (Invitrogen) with OptiMem media. 24h later, cells were lysed using the Dual Glo Luciferase Assay System (Promega) and each 6-well sample was aliquoted into four replicates into a 96 well plate. Renilla luciferase levels were normalized to firefly luciferase. The averages of the three independent experiments were plotted using GraphPad Prism.

### MBP-E6 and E6AP binding assay

The MBP-E6/E6AP binding assay was performed as described [25] with the following modifications. Filter plates (0.45 μm, Millipore, MSHVN4B) were blocked with binding buffer (400 mM NaCl, 50 mM Tris-HCl pH 6.8, 0.1% milk) for 1h and solution was removed prior to addition of MPB-E6 bound amylose beads at indicated concentrations or at 0.67 μM in 50 μl binding buffer with 2 mM DTT. For background subtraction, an amylose bead control was included. MBP bound proteins were blocked in binding buffer at 4°C for 1h. Compounds and DMSO were added in 2 μl per well followed by rotating equilibration for 1h at room temperature. Plates were vacuum filtered and E6AP-BAP (aa 371–440) fusion protein was added at 50 nM in 50 μl of binding buffer with 1 mM DTT. Proteins were incubated for 1-3h at room temperature. Plates were filtered and washed twice with wash buffer (400 mM NaCl, 50 mM Tris-HCl pH 6.8, 0.1% milk) supplemented with 0.1% (v/v) NP40 and once without NP40. Immuno-Star™ AP reagent (Biorad) and enhancer were prepared at a ratio of 1:100 and 60 μl per well added. Reactions developed for 7 min followed by filtering the plate by vacuum. Luminescence was measured in a PHERAstar FS plate reader (BMG Labtech).

### Thermostability assay (TSA)

The thermal stability assay [25, 33] was performed with the following modifications. All proteins were diluted to a final concentration of 1 μM in 400 mM NaCl, 50 mM Tris HCl pH 6.8 and incubated with compounds at the indicated concentrations for 1hr. Compounds were tested at a range of 1 to 150 μM. All compounds were pre-diluted in DMSO resulting a final concentration of 1.8% (v/v) DMSO. Sypro Orange (Invitrogen) was added to a final concentration of 5X. Samples were analyzed in triplicate (25 μL per well) in a 96-well plate using a Real-time PCR (CFX96 Biorad). Fluorescence was measured using the FRET channel of CFX96 Real-Time PCR thermal cycler (BioRad) over a temperature range of 25°C to 95°C. Temperature increments were set to 0.5°C for 30 sec followed by plate reading. In the same fashion, all compounds were evaluated for their fluorescence profile in the absence of MBP-E6. Melt curves and Tm were calculated using Biorad CFX manager software. All proteins were purified three independent times and analysis with compounds was performed at least two times in duplicate per purification run. The average of each individual purification run was determined and these averages of the purifications were plotted using Graph-Pad Prism (v5.00). Results were analyzed by fitting for saturation binding curves to estimate *K*_*d*_ and Bmax values from the best fits. These curves were analyzed in comparison to wild-type using two-way ANOVA with post-hoc Bonferroni analysis for comparison to wild type and a P<0.05 was taken as statistically significant.

### Virtual docking studies

Computational studies were performed using the Schrödinger Maestro software package. The ligands were uploaded to Maestro and ionized (along with desalting and tautomerization) using Epik, in order to generate all possible states within a pH range of 7.0±2.0. The output structures from Epik were then minimized using the OPLS-2005 force field. Starting from a crystal structure of HPV-16 E6 (derived from the original crystal structure 4GIZ, through a set of induced-fit simulations, which allowed for larger ligands to be accommodated by the binding groove), five mutants were generated within Maestro through the Mutate Residues Script (L50G, R10A, R102A, R131A, R55A) [47, 48]. Each mutant was prepared using the Protein Preparation Wizard and minimized in complex with the CAF-40 ligand to an RMSD of 0.30Å, using the OPLS-2005 force field. The CAF-25 and CAF-40 ligands were then flexibly docked into the resulting crystal structure via the induced-fit algorithm, with protein flexibility within 10Å of each ligand. The output structures of the Induced Fit docking runs were further subjected to molecular dynamics (MD) simulations. Solvated models of each mutant and wild-type E6 structure, along with their ligands, were created using Desmond.

### Details of Molecular Docking and Dynamics

E6-ligand complexes were solvated with the TIP3P water model in the presence of 0.15 M sodium chloride buffer. An orthorhombic water box was generated with a 10 Å buffer region, all overlapping water molecules were removed, and the system was neutralized in the presence of sodium cations. The resulting solvated systems were then minimized with the OPLS-2005 force field. Long-range electrostatic interactions were determined using a smooth particle mesh Ewald method, with a grid spacing of 0.8 Å. For non-bonded van der Waals interactions, a cut-off of 9.0 Å was set. All simulations were performed for 5.0 ns using the Desmond NPT method, with a six-step slow relaxation protocol prior to the molecular dynamics run: (**1**) 2000 step limited-memory Broyden-Fletcher-Goldfarb-Shanno (L-BFGS) minimization, with a loose convergence restraint of 50 kcal/mol/Å; (**2**) 2000 step L-BFGS minimization with a convergence constraint of 5 kcal/mol/Å; (**3**) a 12 ps Berendsen NVT simulation at a temperature of 10 K, with restraints on solute heavy atoms; (**4**) a 12 ps Berendsen NPT ensemble at a temperature of 10 K and pressure at 1.01325 bar, with restraints on solute heavy atoms; (**5**) a 24 ps Berendsen NPT ensemble at a temperature of 300 K and a pressure at 1.01325 bar with restraints on solute heavy atoms; (**6**) a 24 ps Berendsen NPT ensemble at a temperature of 300 K and ds< a pressure at 1.01325 bar, with restraints on residues beyond 15 Å of the restrained ligand[49]. The 5.0 ns molecular dynamic simulation run was performed using the NPT ensemble. The temperature of the simulation was kept at 300 K using a Nosé-Hoover thermostat. Pressure was maintained at 1.01325 bar using the Martyna-Tobias-Klein method. Energy and trajectory data were recorded at every 1.2 ps and 5.0 ps, respectively. After completion, simulation data was processed for each structure and convergence was confirmed by measuring the RMSD of protein side chains, heavy atoms, Cα atoms, and ligand conformation, over the course of each simulation. The average trajectories of the final 50 frames for each E6-ligand complex were generated using VMD[50]. Simulation interaction details were obtained using Desmond and structure images were created using Pymol. Finally, the last fifty frames of each MD simulation were averaged and used as a starting structure to re-dock CAF-25 and CAF-40 with the Standard Precision (SP) algorithm. This method does not involve protein flexibility, as it was assumed that the MD ensemble represents the lowest energy conformation of the protein. The docking scores from the SP results were then compared to the reported IC_50_ and TSA data.

The HPV-16 E6 crystal structure used for initial Induced Fit docking was previously generated starting from the published crystal structure (PDB ID: 4GIZ). A long ligand was constructed, capable of spanning the distance between R55 and W132, and it was docked flexibly (Induced Fit) into 4GIZ. The resulting protein structure has a helix groove capable of accepting larger ligands, and is less prone to collapsing on itself. As the E6 protein is predicted to be highly flexible, this structure is an approximation of a more open state of the protein.

## Supporting Information

S1 Table Sequences of primers and their annealing temperatures (Ta) used in this study. (PDF)

S2 Table Summary of the apparent *K*_*d*_ and B_max_ values of CAF-25, -26, -27 and CAF-40 with MBP-E6 wild-type and mutant proteins determined by thermal stability assay. The p53 column summarizes the *in vitro* degradation activity of these proteins. Fit comparison:(*) P<0.05 vs. wild-type (WT)
(PDF)

S1 Fig Characterization of MBP-E6 wild-type and mutant proteins and MD simulations. (A) Analysis of p53 *in vitro* degradation activity of wild-type MBP-E6 in response to concentrations of CAF-26 by western blotting. p53 and MBP-E6 proteins were analyzed by blotting with anti-p53 and anti-E6, respectively. (B) MD simulations of CAF-25 and (C) CAF-40 with E6 protein (PDB ID: 4GIZ). (E) Raw fluorescence melt curves of MBP-E6 wild-type (WT) and mutants (R10A, R55A, R102A, R131A) analyzed using the TSA. (F) Analysis of p53 *in vitro* degradation activity of MBP-E6 wild-type and R102A proteins after 3h incubation. p53 and MBP-E6 (WT, R102A) proteins were analyzed by blotting with anti-p53 and anti-E6, respectively.
(TIF)

S2 Fig ΔTM profile of MBP-E6 mutants in response to CAF-25. ΔTM changes of wild- type (WT) MBP-E6 and (A) R10A, (B) L50G, (C) R55A, and (D) R102A mutant proteins in response to increasing concentrations with CAF-25 subtracted by the DMSO control.
(TIF)

S3 Fig ΔTM profile of MBP-E6 mutants in response to CAF-26. ΔTM changes of wild-type (WT) MBP-E6 and (A) R10A, (B) L50G, (C) R55A, and (D) R102A mutant proteins in response to increasing concentrations with CAF-26 over DMSO control. * P<0.05 compared to WT.
(TIF)

S4 Fig ΔTM profile of MBP-E6 mutants in response to CAF-27. ΔTM changes of wild type (WT) MBP-E6 and (A) R10A, (B) L50G, (C) R55A, (D) R102A and (E) R131A mutant proteins in response to increasing concentrations with CAF-27 over DMSO control. * P<0.05 compared to WT.
(TIF)

S5 Fig ΔTM profile of MBP-E6 mutants in response to CAF-40. ΔTM changes of wild type (WT) MBP-E6 and (A) R10A, (B) L50G, (C) R55A, (D) R102A and (E) R131A mutant proteins in response to increasing concentrations with CAF-40 over DMSO control. * P<0.05 compared to WT.
(TIF)

S6 Fig Molecular dynamics (MD) simulations of CAF-25 with HPV-16 E6 mutants. MD simulations show that R102 and R131 are major contributors to the interaction of CAF-25 with HPV-16 E6. Panels A-E showcase the interactions of various E6 residues with CAF-25 in each respective mutant. Of particular interest are the residues R102 and R131. These two amino acids are main contributors to the interaction between ligand and protein. (E) With the loss of R131, R102 becomes a main driving force in the protein–CAF-25 interaction. (D) When R102 is lost, R129, which has minimal contact with the ligand (A,B,C,E), is shifted to more efficiently interact and results in a change in the shape of the protein (F).
(TIF)

S7 Fig Molecular dynamics (MD) simulations of CAF-40 with HPV-16 E6 mutants. Panels A-E highlight the interactions of various E6 residues with CAF-40 in each respective mutant. The mutations of R131 and R102 cause other rim arginines to move in and aid with the ligand-protein interaction (D,E). Specifically, R102A causes a change in the protein shape to accommodate a more efficient interaction between R129 and CAF-40 (D,F).
(TIF)
