# Supplementary material for: Molecular Probing of the HPV-16 E6 Protein Alpha Helix Binding Groove with Small Molecule Inhibitors
Source: PLoS One. 2016 Feb 25;11(2):e0149845. doi: 10.1371/journal.pone.0149845 (PMC4767726; doi:10.1371/journal.pone.0149845)
Supplement: S1 Table — (PDF) [file pone.0149845.s001.pdf]

Table S1 Sequences of primers and their annealing temperatures (Ta) used in this study.

Mutant	Forward Primer 5'-3'	Reverse Primer 5'-3'	Ta (°C)
F2V	GCCATGGCGATGGTTCAGGACCCAC	GTGGGTCCTGAACCATCGCCATGGC	**61**
R10A	CCCACAGGAGCGACCCGCGAAGTTAC CACAGTTATG	CATAACTGTGGTAACTTCGCGGGTCGCTC CTGTGGG	**63**
L50G	GACTTTGCTTTTCGGGATGGATGCATA GTATATAGAG	CTCTATATACTATGCATCCATCCCGAAAAG CAAAGTC	**55**
R55A	GGGATTTATGCATAGTATATGCAGAT GGGAATCCATATGCTG	CAGCATATGGATTCCCATCTGCATATACTA TGCATAAATCCC	**59**
R102A	CCGTTGTGTGATTTGTTAATTGCGTGT ATTAACTGTCAAAAGCC	GGCTTTTGACAGTTAATACACGCAATTAA CAAATCACACAACGG	**59**
R131A	CATAATATAAGGGGTGCGTGGACCGG TCGATG	CATCGACCGGTCCACGCACCCCTTATATTA TG	**59**
MBP	CTAGTTACGCCATGGCGCCCTGAAAAT AAAGATTCT CGCTCATG-3	GATCCGGTACCGGATCCGAATTCGAGCTC CGTCGACAAGCTTGCG	**66**
