# Supplementary material for: Molecular Probing of the HPV-16 E6 Protein Alpha Helix Binding Groove with Small Molecule Inhibitors
Source: PLoS One. 2016 Feb 25;11(2):e0149845. doi: 10.1371/journal.pone.0149845 (PMC4767726; doi:10.1371/journal.pone.0149845)
Supplement: S2 Table — The p53 column summarizes the in vitro degradation activity of these proteins. Fit comparison:(*) P<0.05 vs. wild-type (WT) (PDF) [file pone.0149845.s002.pdf]

Table S2. Summary of apparent K_d and Bmax values of CAF-25, -26, -27 and CAF-40 with MBP-E6 wild-type and mutant proteins determined by thermal stability assay. The p53 column summarizes the *in vitro* degradation activity of these proteins. Fit comparison:(*) P<0.05 vs. wild-type (WT)

Protein	p53	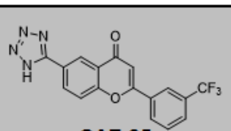 CAF-25		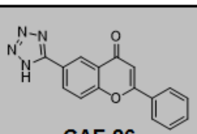 CAF-26		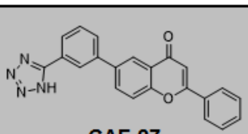 CAF-27		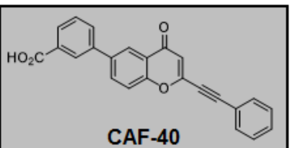 CAF-40	
		Kd (μM)	Bmax	*Kd* (μM)	Bmax	*Kd* (μM)	Bmax	*Kd* (μM)	Bmax
**WT**	**+++**	7.3±1.5	1.25	16.6±4.4	1.42	6.7±1.9	0. 87	5.9±1.7	0.88
**R10A**	**+++**	7.3±1.9	1.48	18.2±5.8	1.77	9.0±3.6	0.96	3.5±1.7	0.79
**L50G**	**-**	7.3±2.1	1.49	13.0±3.3	1.43	3.4±1.3	0.81	11.2±3.9	1.06
**R55A**	**++**	11.7±3.0	1.43	21.7±9.0	1.57	5.5±2.9	0.77	11.7±6.0	0.73
**R102A**	**+/-**	15.3±8.0	1.54	14.2±4.4	1.25	3.2±2.0	**0.62***	**16.5±7.4***	0.73
**R131A**	**+++**	**13.5±2.7***	**1.69***	**9.4± 1.6***	1.43	4.2±1.9	0.79	7.9±3.0	0.97
