# Supplementary figures and images for: Molecular Probing of the HPV-16 E6 Protein Alpha Helix Binding Groove with Small Molecule Inhibitors
Source: PLoS One. 2016 Feb 25;11(2):e0149845. doi: 10.1371/journal.pone.0149845 (PMC4767726; doi:10.1371/journal.pone.0149845)

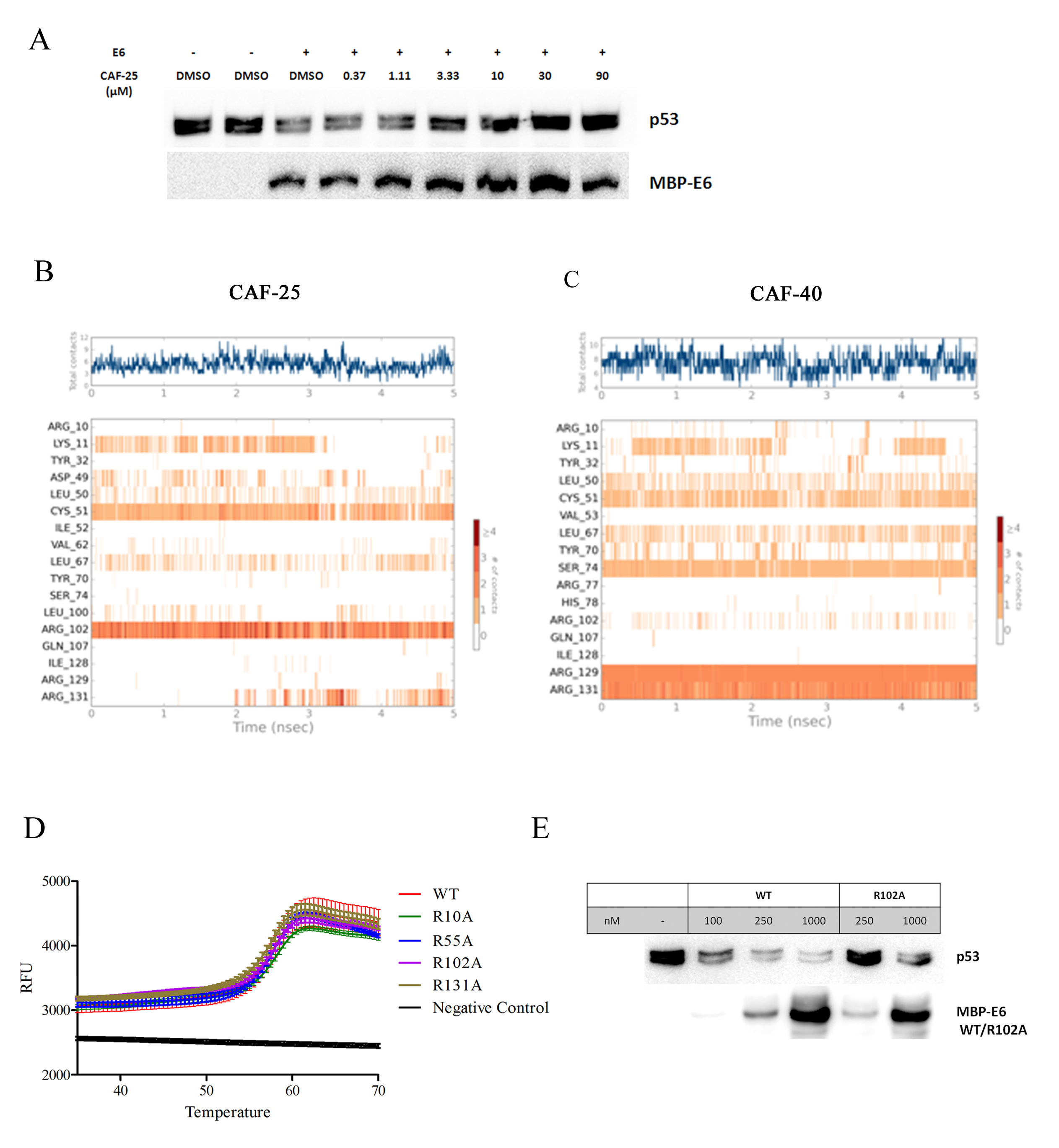

Supplement: S1 Fig — (A) Analysis of p53 in vitro degradation activity of wild-type MBP-E6 in response to concentrations of CAF-26 by western blotting. p53 and MBP-E6 proteins were analyzed by blotting with anti-p53 and anti-E6, respectively. (B) MD simulations of CAF-25 and (C) CAF-40 with E6 protein (PDB ID: 4GIZ). (E) Raw fluorescence melt curves of MBP-E6 wild-type (WT) and mutants (R10A, R55A, R102A, R131A) analyzed using the TSA. (F) Analysis of p53 in vitro degradation activity of MBP-E6 wild-type and R102A proteins after 3h incubation. p53 and MBP-E6 (WT, R102A) proteins were analyzed by blotting with anti-p53 and anti-E6, respectively. (TIF) [file pone.0149845.s003.tif]

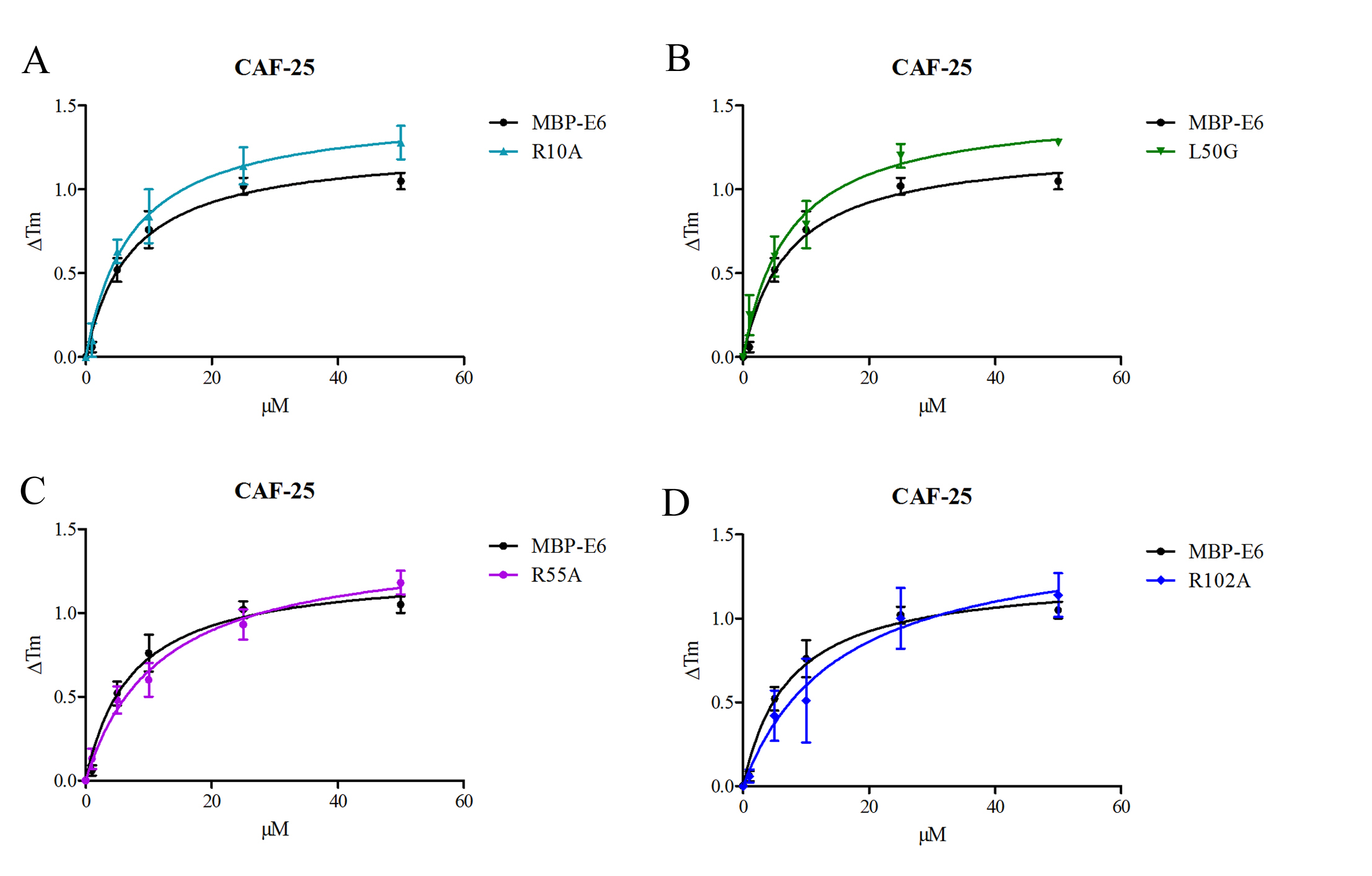

Supplement: S2 Fig — ΔTM changes of wild- type (WT) MBP-E6 and (A) R10A, (B) L50G, (C) R55A, and (D) R102A mutant proteins in response to increasing concentrations with CAF-25 subtracted by the DMSO control. (TIF) [file pone.0149845.s004.tif]

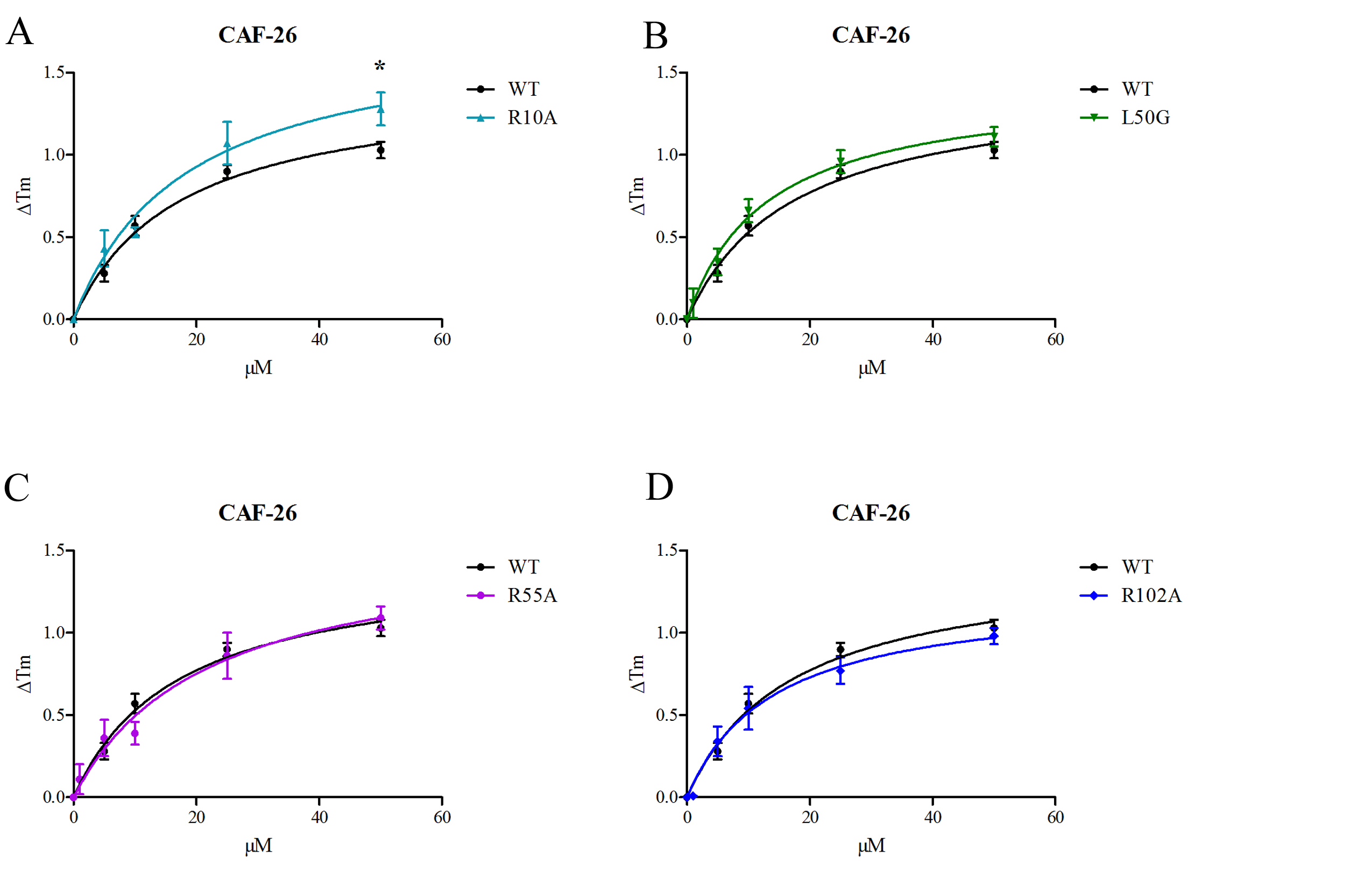

Supplement: S3 Fig — ΔTM changes of wild-type (WT) MBP-E6 and (A) R10A, (B) L50G, (C) R55A, and (D) R102A mutant proteins in response to increasing concentrations with CAF-26 over DMSO control. * P<0.05 compared to WT. (TIF) [file pone.0149845.s005.tif]

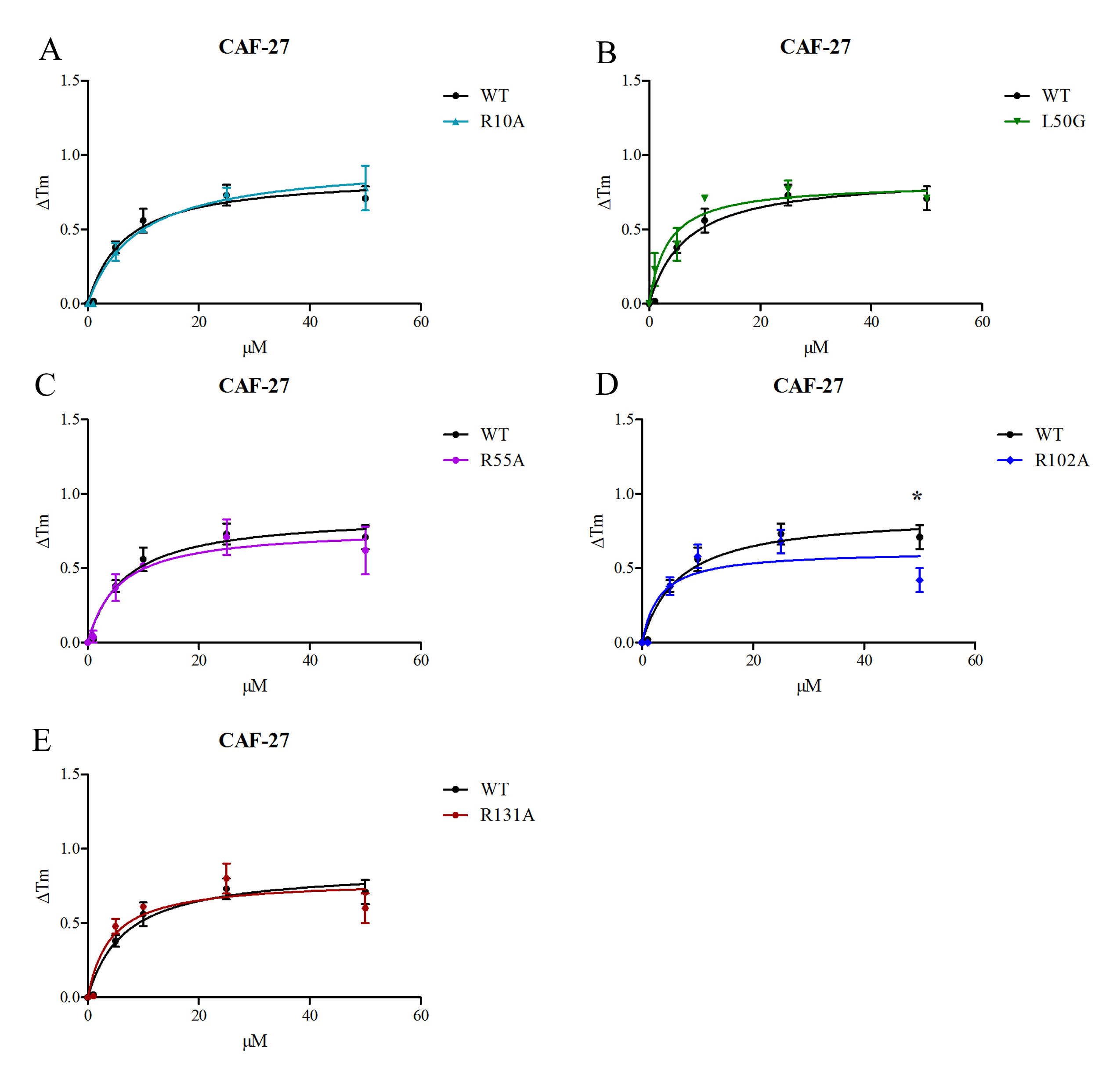

Supplement: S4 Fig — ΔTM changes of wild type (WT) MBP-E6 and (A) R10A, (B) L50G, (C) R55A, (D) R102A and (E) R131A mutant proteins in response to increasing concentrations with CAF-27 over DMSO control. * P<0.05 compared to WT. (TIF) [file pone.0149845.s006.tif]

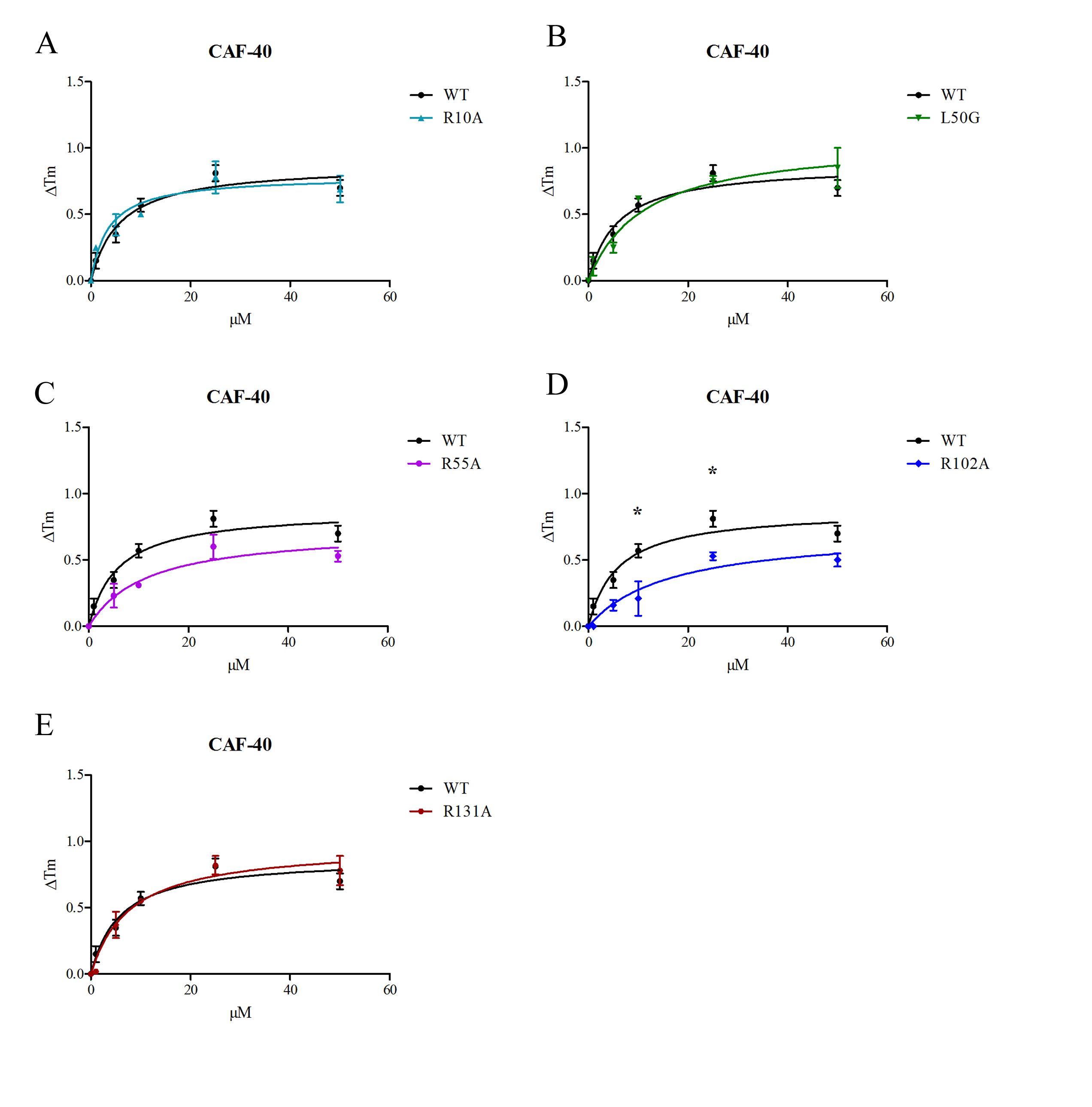

Supplement: S5 Fig — ΔTM changes of wild type (WT) MBP-E6 and (A) R10A, (B) L50G, (C) R55A, (D) R102A and (E) R131A mutant proteins in response to increasing concentrations with CAF-40 over DMSO control. * P<0.05 compared to WT. (TIF) [file pone.0149845.s007.tif]

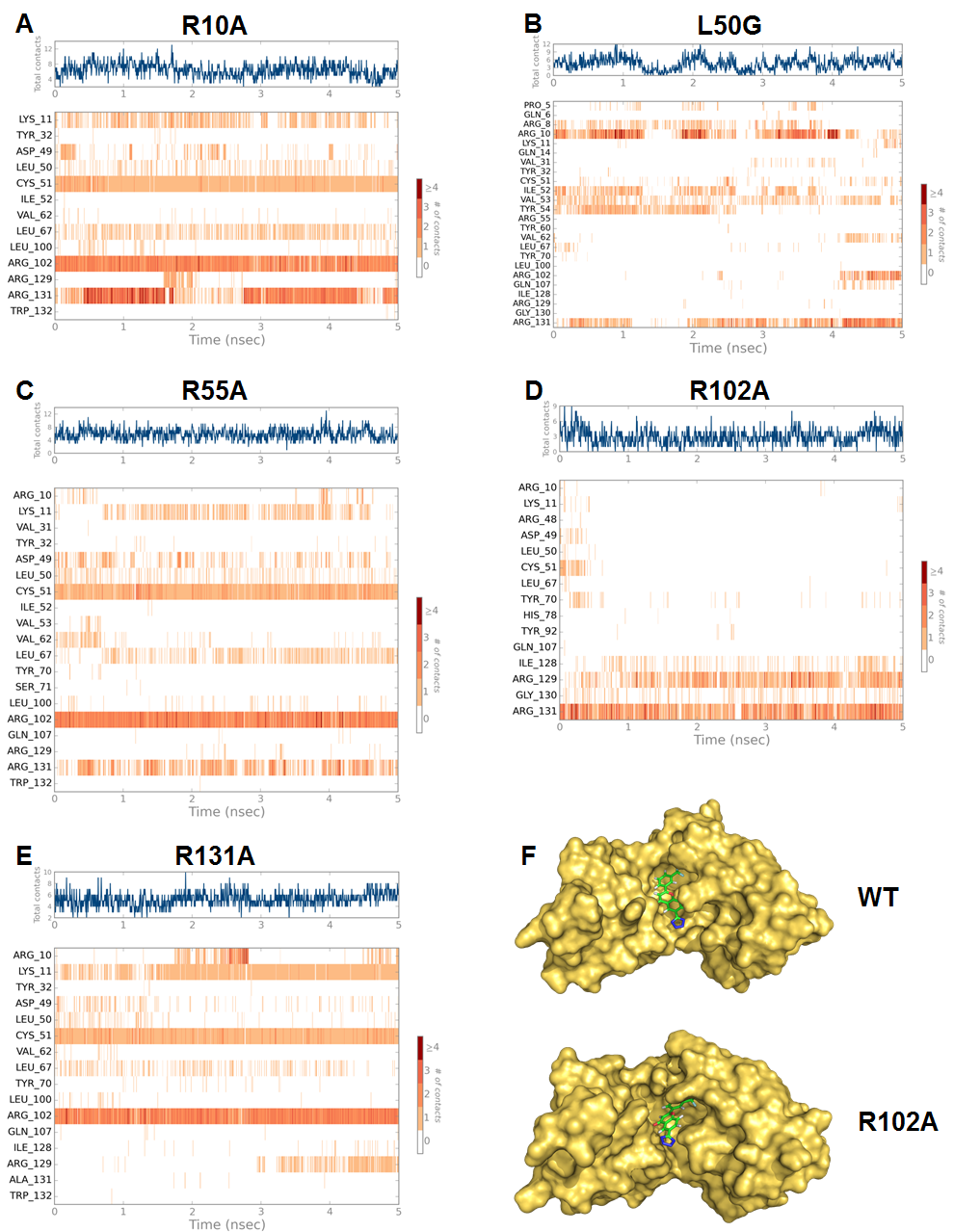

Supplement: S6 Fig — MD simulations show that R102 and R131 are major contributors to the interaction of CAF-25 with HPV-16 E6. Panels A-E showcase the interactions of various E6 residues with CAF-25 in each respective mutant. Of particular interest are the residues R102 and R131. These two amino acids are main contributors to the interaction between ligand and protein. (E) With the loss of R131, R102 becomes a main driving force in the protein–CAF-25 interaction. (D) When R102 is lost, R129, which has minimal contact with the ligand (A,B,C,E), is shifted to more efficiently interact and results in a change in the shape of the protein (F). (TIF) [file pone.0149845.s008.tif]

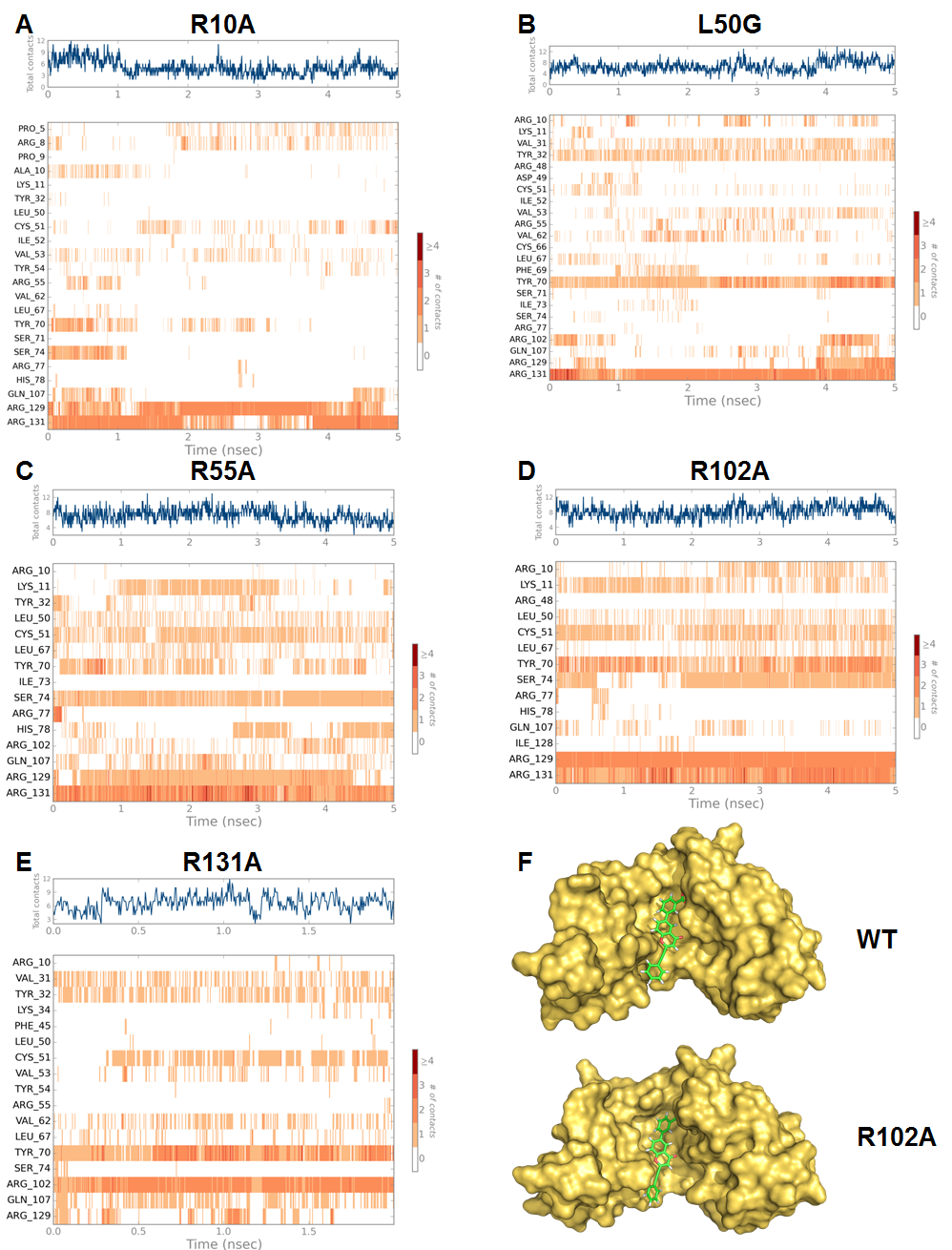

Supplement: S7 Fig — Panels A-E highlight the interactions of various E6 residues with CAF-40 in each respective mutant. The mutations of R131 and R102 cause other rim arginines to move in and aid with the ligand-protein interaction (D,E). Specifically, R102A causes a change in the protein shape to accommodate a more efficient interaction between R129 and CAF-40 (D,F). (TIF) [file pone.0149845.s009.tif]
